# Double sampling of a faecal immunochemical test is not superior to single sampling for detection of colorectal neoplasia: a colonoscopy controlled prospective cohort study

**DOI:** 10.1186/1471-2407-11-434

**Published:** 2011-10-10

**Authors:** Frank A Oort, Sietze T van Turenhout, Veerle MH Coupé, René WM van der Hulst, Eric IC Wesdorp, Jochim S Terhaar sive Droste, Ilhame Ben Larbi, Shannon L Kanis, Edwin van Hengel, Anneke A Bouman, Gerrit A Meijer, Chris JJ Mulder

**Affiliations:** 1 Gastroenterology and Hepatology, VU University Medical Centre, De Boelelaan 1118, Amsterdam, The Netherlands; 2 Epidemiology and Biostatistics, VU University Medical Centre, De Boelelaan 1118, Amsterdam, The Netherlands; 3 Gastroenterology and Hepatology, Kennemer Gasthuis, Boerhaavelaan 22, Haarlem, The Netherlands; 4 Gastroenterology and Hepatology, Sint Lucas Andreas Hospital, Jan Tooropstraat 164, Amsterdam, The Netherlands; 5 Clinical Chemistry, VU University Medical Centre, De Boelelaan 1118, Amsterdam, The Netherlands; 6 Pathology, VU University Medical Centre, De Boelelaan 1118, Amsterdam, The Netherlands

## Abstract

**Background:**

A single sampled faecal immunochemical test (FIT) has moderate sensitivity for colorectal cancer and advanced adenomas. Repeated FIT sampling could improve test sensitivity. The aim of the present study is to determine whether any of three different strategies of double FIT sampling has a better combination of sensitivity and specificity than single FIT sampling.

**Methods:**

Test performance of single FIT sampling in subjects scheduled for colonoscopy was compared to double FIT sampling intra-individually. Test positivity of double FIT sampling was evaluated in three different ways: 1) "one of two FITs+" when at least one out of two measurements exceeded the cut-off value, 2) "two of two FITs+" when both measurements exceeded the cut-off value, 3) "mean of two FITs+" when the geometric mean of two FITs exceeded the cut-off value. Receiver operator curves were calculated and sensitivity of single and the three strategies of double FIT sampling were compared at a fixed level of specificity.

**Results:**

In 124 of 1096 subjects, screen relevant neoplasia (SRN) were found (i.e. early stage CRC or advanced adenomas). At any cut-off, "two of two FITs+" resulted in the lowest and "one of two FITs+" in the highest sensitivity for SRN (range 35-44% and 42%-54% respectively). ROC's of double FIT sampling were similar to single FIT sampling. At specificities of 85/90/95%, sensitivity of any double FIT sampling strategy did not differ significantly from single FIT (p-values 0.07-1).

**Conclusion:**

At any cut off, "one of two FITs+" is the most sensitive double FIT sampling strategy. However, at a given specificity level, sensitivity of any double FIT sampling strategy for SRN is comparable to single FIT sampling at a different cut-off value. None of the double FIT strategies has a superior combination of sensitivity and specificity over single FIT.

## Background

In the United States of America and in Europe, colorectal cancer (CRC) ranks second as cause of cancer related death [1,2]. Screening is the most realistic approach to decrease CRC related mortality. Screening with guaiac-based faecal occult blood tests (g-FOBTs) has been shown to decrease disease specific mortality [3-5]. Faecal immunochemical tests (FITs or i-FOBTs) have been shown to be superior to g-FOBTs [6-9]. A major benefit of (semi-)quantitative FITs is that by adjustment of the threshold for positivity, test characteristics and number of follow-up colonoscopies can be tuned to local resources [10,11]. Since sensitivity of FIT for CRC is in the range of 66-87% [8,12,13], and sensitivity for advanced adenomas is even lower (27-38% depending on the cut-off value) [8,13,14], there is still room for improvement. One approach for improving the sensitivity of FIT based screening could be to increase the number of samples tested, which is common practice for gFOBTs [3-5].

So far, most studies on double FIT sampling either did not perform colonoscopy in FIT negative individuals [15,16], did not evaluate different definitions of positivity for double FIT sampling [14,17,19], or did not assess the effect of different cut-off values [17,20]. In addition, none of these studies evaluated the effect of multiple sampling on specificity.

This prospective, multi-centre cohort study aims to investigate whether sensitivity for the detection of screen relevant neoplasia (CRC stage I, II or advanced adenomas) of single FIT sampling can be increased by double FIT sampling, without substantially affecting specificity. Primary goal is to compare sensitivity and specificity of single FIT sampling and different strategies of double FIT sampling, at a predefined range of cut-off values, in a colonoscopy controlled population. In this study, we report that double and single FIT sampling have a comparable combination of sensitivity and specificity, at a different cut-off value.

## Methods

### Study population

From June 2008 to October 2009, all ambulatory patients (≥18 years) scheduled for elective colonoscopy in three participating medical centres in and around Amsterdam, were invited to participate in this study irrespective of their indication for colonoscopy (i.e. screening, surveillance, or presence of symptoms). Exclusion criteria were either hospitalization, age below 18 years, colostomy, total colectomy, colitis with ulcer(s), or a documented history or subsequent diagnosis of inflammatory bowel disease (IBD). In addition, individuals in which colonoscopic examination remained incomplete due to insufficient bowel lavage or technical difficulties, who did not adhere to the instructions on FIT sampling (e.g. failed to provide the dates of FIT sampling), or could not provide informed consent, were excluded from analysis. The local Medical Ethics Review Boards of each of the hospitals approved this study.

### Study design

All eligible individuals were asked to perform a FIT on two subsequent days prior to colonoscopy. Elective patients were invited to participate in this study by telephone. Individuals interested in the study received a more detailed information package by mail, including two FITs, sampling instructions and an informed consent form. Subjects who could repetitively not be reached by telephone were send the same package with an additional explanatory letter.

An automated FIT was used (OC-sensor^®^, Eiken Chemical Co., Tokyo, Japan). This semi-quantitative test is considered positive when the haemoglobin concentration in the test tube exceeds the pre-determined cut-off value. Patients were instructed to perform this test on two separate days, before bowel preparation by laxatives was started, and write the date of performance on the FIT container.

**Figure 1:**
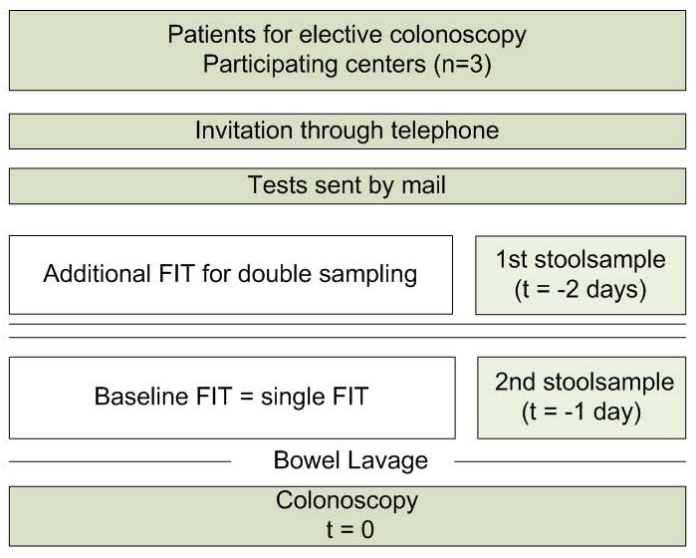
**Study design**. *FIT = faecal immunochemical test*.

The baseline FIT was defined as the sample taken from a bowel movement one day prior to colonoscopy (t = -1), whereas the additional FIT for double sampling was performed on stool produced two days before colonoscopy (t = -2; see Figure 1). Illustrated and written instructions explained participants to sample their stool without contamination with water or urine. All FITs were sampled at home and there were no restrictions in diet or medication during the week in which stool was sampled. Participants were instructed to obtain FIT samples at a maximum of 72 hours prior to colonoscopy, and to put the FIT samples in the zip lock bags that were included in the mail package. Participants were requested to store the zip lock bags in the refrigerator until departure for the endoscopy department.

Completed FITs and informed consent forms were collected at the endoscopy-department at the day of colonoscopy. All FITs were stored at minus 20 degrees Celsius on arrival. Tests were analyzed according to the manufacturer's instructions by an experienced technician, who was unaware of the clinical data, using the OC sensor MICRO desktop analyzer (Eiken Chemical co., Tokyo, Japan) [21].

### Colonoscopy and lesions

All colonoscopies were performed or supervised by experienced gastroenterologists, who were unaware of the FIT results. Patients were offered to take conscious sedation by Midazolam. A complete colonoscopy was defined as intubation of the caecum with identification of the ileocaecal valve or appendiceal orifice, or intubation up to CRC (irrespective of the location and visualisation of the whole colon). Incomplete colonoscopies or colonoscopies with insufficient bowel preparation, as judged by the individual endoscopist, were excluded unless CRC was found. However, if a barium enema, virtual colonography or second colonoscopy was performed within six months, evaluation of the colon was considered complete and the subject was included in analysis. Patients were classified based on the most advanced lesion detected.

Histology of tissue samples obtained was evaluated routinely. Lesion size was estimated by the endoscopist. Adenomas ≥1.0 cm, adenomas with a villous component (i.e. tubulovillous or villous adenoma) or adenomas with high-grade dysplasia were defined as advanced adenomas [22]. Colorectal carcinoma was staged according to the AJCC cancer and TNM staging manual [23]. Screen relevant neoplasia were defined as advanced adenoma and/or early stage cancer (i.e. stage I and II).

### Statistical analysis

Primary outcome measures were sensitivity and specificity of the baseline FIT (t = -1; henceforth single FIT) and three strategies for double FIT sampling (results of t = -1 and t = -2) for the detection of screen relevant neoplasia. Results of single and double sampling were compared intra-individually and colonoscopy and histopathology were considered as gold standard. This study did not have the intention to determine the cut-off value with optimal sensitivity and specificity for screening. Instead, we evaluated whether a combination of sensitivity and specificity for double FIT sampling exists that is superior to single FIT sampling.

Three different strategies for positive reading of double FIT sampling were used:

1. "one of two FITs+": haemoglobin concentrations exceed the cut-off value in at least one out of two samples.

2. "two of two FITs+": haemoglobin concentrations exceed the cut-off value in both samples.

3. "mean of two FITs+": the geometric mean of haemoglobin concentrations from both samples exceeds the cut-off value.

Test sensitivities and specificities were assessed at cut-off values of 50, 75, 100, 150, and 200 ng/ml. The Exact method was used to calculate 95% confidence intervals. Receiver operator curves (ROC's) for detecting screen relevant neoplasia were calculated for single FIT and all three strategies of double FIT sampling. In addition, sensitivities of all three strategies for double FIT sampling were compared to single FIT sampling at a specificity of 85%, 90% and 95% using McNemar's test for correlated proportions. All analyses were performed with SPSS for Windows Version 15.0 (SPSS Inc., Chicago, USA).

## Results

### Participants

**Figure 2:**
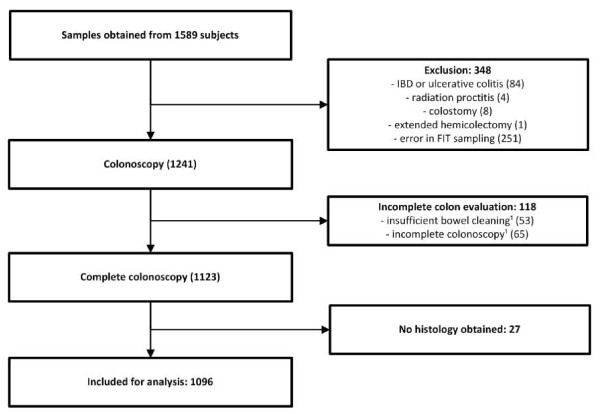
**Study flow diagram of 1589 subjects who participated in FIT sampling and subsequently underwent colonoscopy**. ¹Incomplete colon evaluation in spite of possible additional evaluation by repeated colonoscopy, barium enema or virtual colonography. *FIT = faecal immunochemical test, IBD = inflammatory bowel disease*.

Samples were returned by 1589 patients, 493 of which were excluded from further analysis because of reasons listed in Figure 2. In 33 cases repeated colonoscopy or radiology was performed. Mean age of the participants included was 60, 0 years (range 19-91 yrs, SD 12.5) and 48% of the study cohort was male.

Table 1 shows the primary indications for colonoscopy in individuals eligible for analysis. In this cohort 59% (N = 646) of individuals were referred for colonoscopy because of symptoms, whereas 37% (N = 408) of subjects were referred for screening or surveillance colonoscopy. In 4% (N = 42) of all individuals the indication remained unspecified.

**Table 1:** Primary indications for colonoscopy among 1096 consecutive patients enrolled for evaluation of double FIT sampling

Indication Group Symptomatic/suspect	Indication for colonoscopy	N
	Weight loss	11
	Clinical suspicion of diverticulitis	7
	Clinical suspicion of IBD	8
	
	Abdominal pain	110
	
	Anaemia	71
	Hematochezia	156
	Altered bowel habits	182
	Clinical or radiological suspicion of CRC	25
	Colonoscopy for polypectomy	21
	Diarrhoea	31
	Constipation	24
	**Total**	**646**
**Screening & Surveillance**	Average risk	39
	Familial history of CRC	111
	Lynch syndrome	17
	Polyp surveillance	196
	Post CRC surveillance	45
	**Total**	**408**
**Other**	Not specified/others	**42**
**Grand total**		**1096**

FIT = faecal immunochemical test, IBD = inflammatory bowel disease, CRC = colorectal cancer

### Colonoscopy results

Colorectal cancer was found in 35 (3, 2%) of 1096 included individuals. Malignancies were classified as stage I in 7 (20%), stage II in 13 (37%), stage III in 6 (17%) and stage IV in 3 (9%) patients. Six rectal cancers (17%) could not be staged accurately due to the effects of preoperative radiation. In 104 (9, 5%) individuals, one or more advanced adenomas were found. Consequently, screen relevant neoplasia were found in 124 (11, 3%) subjects.

### Colorectal neoplasia detection and positivity rates

At a cut-off value of 50 ng/ml, the positivity rate of single FIT was 17%, resulting in detection of 91, 4% (32/35) of CRCs and 60, 6% (63/104) of all advanced adenomas found at colonoscopy. In subjects who tested negative for occult blood on single FIT, the additional FIT detected 2 more CRCs and 7 additional advanced adenomas.

Positivity rates ranged from 17-10% (with increasing cut-off values) for single FIT, from 22-12% for "one of two FITs+", from 12-7% for "two of two FITs+", and from 17-9% for "mean of two FITs+".

### Sensitivity and specificity of single and double FIT strategies

Performance characteristics of single FIT and different strategies of double FIT sampling for detecting screen relevant neoplasia, at different cut-off values, are shown in table 2.

**Table 2:** Test characteristics of single and double FIT sampling for detection of screen relevant neoplasia

	Single FIT		"one of two FITs+"		"two of two FITs+"		"mean of two FITs+"	
**Cut-off value**	**Sens**	**Spec**	**Sens**	**Spec**	**Sens**	**Spec**	**Sens**	**Spec**

**Cut-off 50**	**47, 6%**	**88, 2%**	**54, 0%**	**83, 0%**	**43, 5%**	**93, 2%**	**52, 4%**	**88, 8%**
**N**	59/124	844/957	67/124	794/957	54/124	892/957	65/124	850/957
**(CI)**	(39-57)	(86-90)	(45-63)	(80-85)	(35-53)	(91-95)	(43-61)	(87-91)

**Cut-off 75**	**46, 0%**	**90, 5%**	**52, 4%**	**87, 4%**	**41, 1%**	**94, 4%**	**46, 8%**	**91, 3%**
**N**	57/124	866/957	65/124	836/957	51/124	903/957	58/124	874/957
**(CI)**	(37-55)	(88-92)	(43-61)	(85-89)	(32-50)	(93-96)	(38-56)	(89-93)

**Cut-off 100**	**45, 2%**	**92, 5%**	**51, 6%**	**89, 8%**	**39, 5%**	**95, 5%**	**44, 4%**	**92, 8%**
**N**	56/124	885/957	64/124	859/957	49/124	914/957	55/124	888/957
**(CI)**	(36-54)	(91-94)	(42-61)	(88-92)	(31-49)	(94-9s7)	(35-54)	(91-94)

**Cut-off 150**	**42, 7%**	**94, 6%**	**47, 6%**	**92, 2%**	**35, 5%**	**96, 8%**	**38, 7%**	**94, 1%**
**N**	53/124	905/957	59/124	882/957	44/124	926/957	48/124	901/957
**(CI)**	(34-52)	(93-96)	(39-57)	(89-93)	(27-45)	(95-98)	(30-48)	(92-96)

**Cut-off 200**	**37, 9%**	**95, 1%**	**41, 9%**	**93, 2%**	**34, 7%**	**97, 5%**	**37, 1%**	**96, 1%**
**N**	47/124	910/957	52/124	892/957	43/124	933/957	46/124	920/957
**(CI)**	(29-47)	(94-96)	(39-57)	(91-95)	(26-44)	(96-98)	(29-46)	(95-97)

Test characteristics at different cut-off values (ng/ml) of single and three strategies of double FIT sampling for detection of screen relevant neoplasia in 1081* individuals referred for colonoscopy (SRN in 124, no AA nor CRC in 957). *15 cases of late stage CRC were excluded since they were not considered screen relevant.

FIT = faecal immunochemical test, SRN = screen relevant neoplasia, AA = advanced adenoma, CRC = colorectal cancer, "one of two FITs+" = at least one of both FITs above the cut-off value, "two of two FITs+" = both FITs above the cut-off value, "mean of two FITs+" = geometric mean of both FITs above the cut-off value, CI = confidence interval, sens = sensitivity, spec = specificity

At each cut-off value, maximum sensitivity for screen relevant neoplasia was obtained with "one of two FITs+". Compared to single FIT, the highest increase in sensitivity was obtained with "one of two FITs+" at either 50, 75 or 100 ng/ml (6.4% increase over single FIT). However, the confidence intervals of the sensitivity of single FIT and "one of two FITs+" overlapped, and the specificity of "one of two FITs+" (83.0, 87.4 and 89.8% at 50, 75, and 100 ng/ml, respectively) was lower than for single FIT (88.2, 90.5, and 92.5% at 50, 75, and 100 ng/ml, respectively).

At each cut-off value, maximum specificity was found with "two of two FITs+". The highest specificity (97, 5%) of all double FIT strategies was observed for "two of two FITs+" at the highest cut-off value (200 ng/ml). However, "two of two FITs+" resulted in lower sensitivities than single FIT. Moreover, by using single FIT, comparable specificities as for "two of two FITs+" could be reached (up to 95%) by using higher cut-off values (see table 2).

Test characteristics of double FIT sampling strategies were comparable to single FIT sampling at a different cut-off value. For example at 75 ng/ml, the sensitivity of "one of two FITs+" (52%) was higher than the sensitivity of single FIT (46%). However, when the cut-off value of single FIT was decreased to 50 ng/ml, sensitivity became 48% (CI 39-57) which is close to sensitivity of "one of two FITs+" (52%; CI 43-61). The accompanying specificity of single FIT at 50 ng/ml (88, 2%) was virtually equivalent to the specificity of "one of two FITs+" (87, 4%). As shown in table 2, test characteristics of "mean of two FITs+" were comparable to single FIT.

### Receiver operator curves

**Figure 3:**
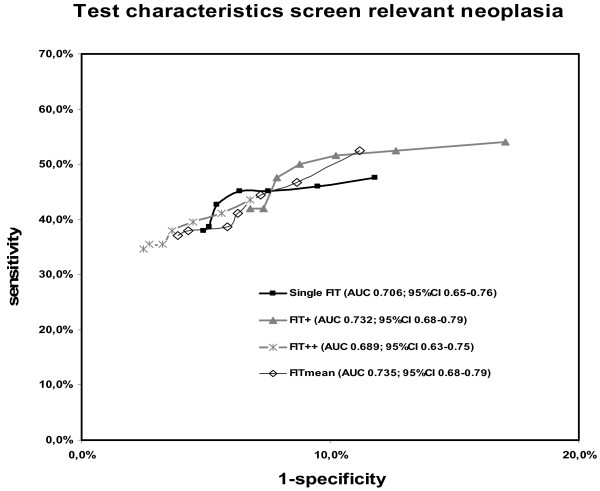
**ROC curves of single and double FIT sampling strategies for the detection of screen relevant neoplasia**. *FIT = faecal immunochemical test, "one of two FITs+" = at least one of both FITs above the cut-off value, "two of two FITs+" = both FITs above the cut-off value, "mean of two FITs+" = geometric mean of both FITs above the cut-off value, AUC = area under the curve, CI = confidence interval*.

For single FIT and the three double FIT strategies, ROC's were constructed (see Figure 3). Highest sensitivities were reached with "one of two FITs+" and "mean of two FITs+", whereas the highest specificities were reached with "two of two FITs+". For all double FIT strategies, ROC's and area under the curves (AUC's) either overlapped or were very close to each other (see Figure 3). Although the highest AUC was found for "mean of two FITs+", all AUC's were within the 95% confidence interval of the AUC of single FIT.

### Comparison at fixed specificities

To evaluate to what extent an increase in sensitivity by double FIT sampling went at the cost of decreased specificity, single FIT and the three double FIT strategies were analyzed at equal specificities. Table 3 shows cut-off values and sensitivities at 85%, 90% and 95% specificity, for each strategy. At any of these specificities, no strategy for double FIT sampling yielded a sensitivity that differed significantly from the sensitivity of single FIT.

**Table 3:** Comparison of sensitivity of single and double FIT sampling for screen relevant neoplasia, at fixed specificities

	Single FIT		"one of two FITs+"			"two of two FITs+"			"mean of two FITs+"		
**Spec**	**Sens**	**Cut-off**	**Sens**	**Cut-off**	**p-value**	**Sens**	**Cut-off**	**p-value**	**Sens**	**Cut-off**	**p-value**

**85%**	51, 6%	34	53, 2%	59	1	n.a.*	n.a.*	n.a.*	53, 2%	40	1

**90%**	46, 0%	73	51, 6%	103	0, 07	44, 4%	27	0, 687	50, 8%	60	0, 125

**95%**	38, 7%	184	37, 9%	371	1	41, 1%	91	0, 453	37, 9%	159	1

Corresponding cut-off values (ng/ml) and sensitivities for screen relevant neoplasia of single FIT sampling and three different strategies of double FIT sampling at fixed specificities of 85%, 90% and 95%.

FIT = faecal immunochemical test, "one of two FITs+" = at least one of both FITs above the cut-off value, "two of two FITs+" = both FITs above the cut-off value, "mean of two FITs+" = geometric mean of both FITs above the cut-off value, spec = specificity, sens = sensitivity, cut-off = cut-off value, n.a.* = no corresponding sensitivity and cut-off value found in the range 50-200 ng/ml

### Additional analysis

All analyses described above were repeated for the outcomes advanced adenomas and CRC. Results are shown in additional file 1: tables S1-S4 and additional file 1: figures S1 and S2. The results found were very similar to those for screen relevant neoplasia.

In total 251 cases were excluded because of an error in FIT sampling. The majority of 155 cases was excluded as the date of sampling of one or both of the tests was unsure. These cases were included in additional analysis, to evaluate if exclusion of these cases would cause bias. As shown in the Additional file 1, the results of these analysis were similar. The remaining 96 sampling errors were due to sampling on or after the day of colonoscopy, performance of only one test, or failure in FIT analysis.

## Discussion

In the present study three different strategies of double FIT sampling were compared to single FIT sampling. In total, 1096 subjects were included and evaluated by colonoscopy. None of the double FIT strategies proved to have a superior combination of sensitivity and specificity compared to single FIT sampling, as is clear from the comparable ROC's and similar AUC's found for all strategies. When comparing sensitivities of single FIT and the three double FIT strategies at fixed specificities of 85%, 90% and 95%, no relevant differences were observed. In fact, at every level of specificity, a comparable sensitivity as observed for "one of two FITs+" could be obtained by single FIT by simply lowering the cut off value.

A priori expectations were that double FIT sampling would increase sensitivity, as this has been observed previously for g-FOBT and FIT [18,19,24]. Accordingly, it was shown in the present study that the highest sensitivity was obtained for ''one of two FITs+''. However, this strategy resulted in the lowest specificity.

Our findings are in line with a recent study in a population with an increased risk for CRC, in which AUC's for the highest out of one, two or three FITs did not differ [18]. Although a direct comparison with a recent Italian screening study is difficult due to different methodology, the authors could also not find a clear superior performance of double over single FIT sampling either [15]. Two other studies on double FIT sampling lacked calculation of direct sensitivity and specificity, as colonoscopy was not performed in FIT negative individuals [15,16]. These characteristics are needed to determine how an increase in sensitivity is counterbalanced by a decrease in specificity. Less recent studies did not use quantitative FITs or did not evaluate test characteristics at different cut-off values [17,20]. In a recent study with a high CRC prevalence, average risk individuals sampled stool before screening colonoscopy. The authors found that the sensitivity increased and specificity decreased when a lower cut-off value or multiple tests were used. However, no comparison was made at an equal specificity. The AUC's for advanced neoplasia for one, two or three FITs did not differ [19]. In the present study, the full potential of double FIT sampling was further studied by evaluation of several definitions of positivity. The present study adds important information as it is the first to determine if any of three strategies of double FIT sampling could increase sensitivity for screen relevant neoplasia, without substantially affecting specificity, at different cut-off values and in a colonoscopy controlled population.

A limitation of the present study is that not a screening population was tested but a referral population, partially containing high risk individuals. Therefore, test characteristics that depend on the prevalence of disease, i.e. positive and negative predictive values, cannot be generalized from this study to the screening population. However, the present study focused on sensitivity and specificity, test characteristics that are not influenced by the prevalence of the disease [25]. Still, in this referral population, sensitivity may be overestimated and specificity underestimated due to work-up bias [26]. This may occur as symptomatic participants have an increased likelihood for having both a positive FIT and a colorectal neoplasm. In particular, it should be noted that lower sensitivities for FIT in a screening population have been reported [19,27]. On the other hand, we carried out a formal comparison of FIT results in CRC cases from a screening and referral cohort and found similar FIT results after correcting for tumour stage [28]. Since for screening, only early stage cancers are relevant, in the present study late stage cancers were excluded from the analysis. Although possible differences in FIT results between referral populations, like in the present study, and screening populations cannot fully be excluded, the present study design still allows for comparing the sensitivities of different sampling schemes for FIT for early stage colorectal cancer. However, a complete correction of work-up bias cannot be ascertained. One should keep in mind that alternative study designs also have limitations like absence of a gold standard because no colonoscopies were performed, or in case colonoscopies were performed, relatively low numbers of cancers found [15,16,27]. In addition, in many studies different FITs, different endpoints (advanced adenoma, advanced neoplasia, screen relevant neoplasia), a different amount of cases, and a different selection of participants (e.g. subjects participating in colonoscopy screening) are used.

To evaluate the effect of work-up bias, analyses were repeated after exclusion of subjects with rectal blood loss, anaemia and clinical suspicion of CRC (data not shown). Although the sensitivities for advanced adenomas found were 4.5-10% lower, our results were similar in the sense that double FIT sampling did not yield any superior combination of sensitivity and specificity compared to single FIT. For CRC data were similar, although too few cases remained to draw firm conclusions (data not shown).

In the current study the number of excluded participants was relatively high. This was mainly due to our stringent protocol on FIT sampling. Of the 251 individuals that were excluded from further analysis, in the majority of cases this was because date of sampling was not registered correctly on the FIT container, as described in the study protocol. Additional analysis including these cases showed similar results. The percentage of incomplete colonoscopies in the present study is in line with previous studies [7,29].

According to our study protocol all FITs should be stored in the refrigerator close to the moment of handing in. In addition, both FITs are sampled maximum 72 hours prior to colonoscopy. As such, the time that the tests are at room temperature is kept as limited as possible. FITs kept at higher temperatures, are more susceptible to a decrease in sensitivity as a result of haemoglobin degradation. When compared to at least one of the screening studies [7] this is still a relative short period of time. Therefore, only a slight decrease in haemoglobin concentration is to be expected [30].

An important asset of the present study is the relatively high tumour yield, which allowed analyzing FIT performance for early and late stage CRC separately. As the potential health gain is highest for individuals with early stage cancer [31], this is relevant for population based screening programs. A second strength of this study is the fact that colonoscopy results were available for all participants, allowing the direct calculation of sensitivities and specificities.

## Conclusion

In conclusion, this study strongly suggests that double FIT sampling, regardless of the definition of test positivity, does not provide a superior combination of sensitivity and specificity compared to single FIT sampling. Moreover, if it is aimed to increase sensitivity at the cost of specificity, this can be achieved equally well by lowering the cut-off value of single FIT sampling rather than by double FIT sampling. To what extent these findings pertain to the general population awaits confirmation in a screening setting.

## Competing interests

All authors have no potential financial, professional or personal conflicts by publishing this manuscript.

S.T. van Turenhout was supported by a research grand from CTMM (Centre for Translational Molecular Medicine), The Netherlands. This company had no influence on any aspect relevant to this study.

The OC sensor MICRO desktop analyzer was provided by Eiken Chemical co., Tokyo, Japan. This company had no influence on any aspect relevant to this study.

## Authors' contributions

FAO and STVT participated in the study concept and design; acquisition of data; analysis and interpretation of data, statistical analysis and drafting of the manuscript. VMHC participated in the study concept and design; statistical analysis and interpretation of data and critical revision of the manuscript for important intellectual content. RWMVDH participated in the study concept and design; acquisition of data and critical revision of the manuscript for important intellectual content. EW participated in the study concept and design and acquisition of data. JSTSD participated in acquisition and interpretation of data, and critical revision of the manuscript for important intellectual content. IBL and SLK participated in acquisition and interpretation of data. EVH and AAB participated in acquisition and interpretation of data, technical and material support. GAM and CJJM participated in the study concept and design; acquisition of data; analysis and interpretation of data, critical revision of the manuscript for important intellectual content, and study supervision. All authors read and approved the final manuscript.

## Pre-publication history

The pre-publication history for this paper can be accessed here:

http://www.biomedcentral.com/1471-2407/11/434/prepub

## Supplementary Material

Additional file 1 **Data on colorectal cancer and advanced adenomas**. Click here for file
