# Supplementary material for: Double sampling of a faecal immunochemical test is not superior to single sampling for detection of colorectal neoplasia: a colonoscopy controlled prospective cohort study
Source: BMC Cancer. 2011 Oct 10;11:434. doi: 10.1186/1471-2407-11-434 (PMC3201938; doi:10.1186/1471-2407-11-434)
Supplement: Additional file 1 — Data on colorectal cancer and advanced adenomas. [file 1471-2407-11-434-S1.RTF]

Additional files
Table I Test characteristics of single and double FIT sampling for detection of colorectal cancer.
	Single FIT		“one of two FITs+”		“two of two FITs+”		“mean of two FITs+”		
Cut-off value	Sens	Spec	Sens	Spec	Sens	Spec	Sens	Spec	
Cut-off 50
N
(CI)	91,4%
32/35
(77-98)	85,5%
907/1061
(83-88)	97,1%
34/35
(85-100)	80,1%
850/1061
(78-82)	91,4%
32/35
(77-98)	90,5%
960/1061
(89-92)	97,1%
34/35
(85-100)	85,6%
908/1061
(83-88)	
Cut-off 75
N
(CI)	91,4%
32/35
(77-98)	87,7%
931/1061
(86-90)	97,1%
34/35
(85-100)	84,3%
894/1061
(82-86)	91,4%
32/35
(77-98)	91,8%
974/1061
(90-93)	97,1%
34/35
(85-100)	88,5%
939/1061
(86-90)	
Cut-off 100
N
(CI)	91,4%
32/35
(77-98)	89,6%
951/1061
(88-91)	97,1%
34/35
(85-100)	86,5%
918/1061
(84-89)	85,7%
30/35
(70-95)	92,9%
986/1061
(91-94)	94,3%
33/35
(81-99)	90,0%
955/1061
(88-92)	
Cut-off 150
N
(CI)	88,6%
31/35
(73-97)	91,8%
974/1061
(90-93)	97,1%
34/35
(85-100)	89,2%
946/1061
(87-91)	80,0%
28/35
(63-92)	94,5%
1003/1061
(93-96)	94,3%
33/35
(81-99)	91,9%
975/1061
(90-93)	
Cut-off 200
N
(CI)	88,6%
31/35
(73-97)	92,8%
985/1061
(91-94)	94,3%
33/35
(81-99)	90,8%
963/1061
(89-92)	80,0%
28/35
(63-92)	95,3%
1011/1061
(94-96)	88,6%
31/35
(73-97)	93,9%
996/1061
(92-95)	
		Test characteristics for different cut-off values (ng/ml) in three strategies of double FIT sampling, in comparison to single FIT sampling for detection of colorectal cancer in 1096 individuals referred for colonoscopy (CRC in 35, no CRC in 1061).	
		FIT = faecal immunochemical test, CRC = colorectal cancer, “one of two FITs+” = at least one of both FITs above the cut-off value, “two of two FITs+” = both FITs above the cut-off value, “mean of two FITs+” = geometric mean of both FITs above the cut-off value, CI = confidence interval, sens = sensitivity, spec = specificity

Table II. Test characteristics of single and double FIT sampling for detection of advanced adenomas.
	Single FIT		“one of two FITs+”		“two of two FITs+”		“mean of two FITs+”		
Cut-off value	Sens	Spec	Sens	Spec	Sens	Spec	Sens	Spec	
Cut-off 50
N
(CI)	39,4%
41/104
(30-49)	88,2%
844/957
(86-90)	46,2%
48/104
(36-56)	83,0%
794/957
(80-85)	34,6%
36/104
(26-45)	93,2%
892/957
(91-95)	44,2%
46/104
(35-54)	88,8%
850/957
(87-91)	
Cut-off 75
N
(CI)	37,5%
39/104
(28-48)	90,5%
866/957
(88-92)	44,2%
46/104
(35-54)	87,4%
836/957
(85-89)	31,7%
33/104
(23-42)	94,4%
903/957
(93-96)	37,5%
39/104
(28-48)	91,3%
874/957
(89-93)	
Cut-off 100
N
(CI)	36,5%
38/104
(27-47)	92,5%
885/957
(91-94)	43,3%
45/104
(34-53)	89,8%
859/957
(88-92)	30,8%
32/104
(22-41)	95,5%
914/957
(94-97)	35,6%
37/104
(26-46)	92,8%
888/957
(91-94)	
Cut-off 150
N
(CI)	33,7%
35/104
(25-44)	94,6%
905/957
(93-96)	38,5%
40/104
(29-49)	92,2%
882/957
(89-93)	26,0%
27/104
(18-35)	96,8%
926/957
(95-98)	28,8%
30/104
(20-39)	94,1%
901/957
(92-96)	
Cut-off 200
N
(CI)	27,9%
29/104
(20-38)	95,1%
910/957
(94-96)	31,7%
33/104
(23-42)	93,2%
892/957
(91-95)	25,0%
26/104
(17-34)	97,5%
933/957
(96-98)	26,9%
28/104
(19-37)	96,1%
920/957
(95-97)	
		Test characteristics for different cut-off values (ng/ml) in three strategies of double FIT sampling, in comparison to single FIT sampling for detection of advanced adenomas in 1061* individuals referred for colonoscopy (AA in 104, no AA nor CRC in 957). *cases of CRC (N=35) were excluded for calculation of specificity.	
		FIT = faecal immunochemical test, AA = advanced adenoma, CRC = colorectal cancer, “one of two FITs+” = at least one of both FITs above cut-off the value, “two of two FITs+” = both FITs above the cut-off value, “mean of two FITs+” = geometric mean of both FITs above the cut-off value, CI = confidence interval, sens = sensitivity, spec = specificity
Figure I. ROC curves of single and double FIT sampling strategies for the detection of colorectal cancer.

		FIT = faecal immunochemical test, CRC = colorectal cancer, “one of two FITs+” = at least one of both FITs above the cut-off value, “two of two FITs+” = both FITs above the cut-off value, “mean of two FITs+” = geometric mean of both FITs above the cut-off value, AUC = area under the curve, CI = confidence interval 


Figure II. ROC curves of single and double FIT sampling strategies for the detection of advanced adenomas.

		FIT = faecal immunochemical test, “one of two FITs+” = at least one of both FITs above the cut-off value, “two of two FITs+” = both FITs above the cut-off value, “mean of two FITs+” = geometric mean of both FITs above the cut-off value, AUC = area under the curve, CI = confidence interval

Table III. Comparison of sensitivity of single and double FIT sampling for colorectal cancer, at fixed specificities.
	Single FIT		“one of two FITs+”			“two of two FITs+”			“mean of two FITs+”			
Spec	Sens	Cut-off	Sens	Cut-off	p-value	Sens	Cut-off	p-value	Sens	Cut-off	p-value	
85%	91,4%	46	97,1%	79	0,5	n.a.*	n.a.*	n.a.*	97,1%	48	0,5	
90%	91,4%	110	94,3%	164	1	91,4%	41	1	94,3%	101	1	
95%	82,9%	404	82,9%	828	1	80,0%	174	1	88,6%	274	0,5	

		Corresponding cut-off values (ng/ml) and sensitivities for CRC of single FIT sampling and three different strategies of double FIT sampling at fixed specificities.	
		FIT = faecal immunochemical test, CRC = colorectal cancer, “one of two FITs+” = at least one of both FITs above the cut-off value, “two of two FITs+” = both FITs above the cut-off value, “mean of two FITs+” = geometric mean of both FITs above the cut-off value, spec = specificity, sens = sensitivity, cut-off = cut-off value,  n.a.* = no corresponding sensitivity and cut-off value found in the range 50-200ng/ml 
Table IV. Comparison of sensitivity of single and double FIT sampling for advanced adenomas, at fixed specificities.
	Single FIT		“one of two FITs+”			“two of two FITs+”			“mean of two FITs+”			
Spec	Sens	Cut-off	Sens	Cut-off	p-value	Sens	Cut-off	p-value	Sens	Cut-off	p-value	
85%	44,2%	34	45,2%	59	0,774	n.a.*	n.a.*	n.a.*	45,2%	40	0,774	
90%	37,5%	73	43,3%	103	0,039†	35,6%	27	0,687	42,3%	60	0,07	
95%	28,8%	184	27,9%	371	1	31,7%	91	0,453	27,9%	159	1	

Corresponding cut-off values (ng/ml) and sensitivities for advanced adenomas of single FIT sampling and three different strategies of double FIT sampling at fixed specificities.  	
FIT = faecal immunochemical test, “one of two FITs+” = at least one of both FITs above the cut-off value, “two of two FITs+” = both FITs above the cut-off value, “mean of two FITs+” = geometric mean of both FITs above the cut-off value, spec = specificity, sens = sensitivity, cut-off = cut-off value,  n.a.* = no corresponding sensitivity and cut-off value found in the range 50-200ng/ml. †: not corrected for multiple comparisons

Table V Test characteristics of single and double FIT sampling for detection of screen relevant neoplasia, including 251 cases with unsure date of FIT sampling.
	Single FIT		“one of two FITs+”		“two of two FITs+”		“mean of two FITs+”		
Cut-off value	Sens	Spec	Sens	Spec	Sens	Spec	Sens	Spec	
Cut-off 50
N
(CI)	51,0%
75/147
(43-59)	87,8%
955/1088
(86-90)	57,8%
85/147
 (50-66)	82,4%
897/1088
 (80-85)	46,9%
69/147
 (39-55)	92,6%
1008/1088
 (91-94)	56,5%
83/147
 (48-56)	88,1%
959/1088
 (86-90)	
Cut-off 75
N
(CI)	48,3%
71/147
 (40-57)	90,3%
982/1088
 (83-92)	56,8%
82/147
 (47-64)	86,9%
945/1088
 (85-89)	44,5%
64/147
 (35-52)	94,2%
1025/1088
 (93-96)	51,0%
75/147
 (43-59)	90,7%
987/1088
 (89-92)	
Cut-off 100
N
(CI)	47,6%
70/147
 (39-56)	92,2%
1003/1088
 (90-94)	55,1%
81/147
 (47-63)	89,2%
971/1088
 (87-91)	40,8%
60/147
 (33-49)	95,3%
1037/1088
 (94-96)	47,6%
70/147
 (39-56)	92,4%
1005/1088
 (91-94)	
Cut-off 150
N
(CI)	44,9%
61/147
 (33-50)	94,1%
1024/1088
(93-95)	51,0%
75/147
 (43-49)	91,5%
996/1088
 (90-93)	36,7%
54/147
 (29-45)	96,4%
1049/1088
 (95-97)	42,2%
62/147
 (34-51)	93,8%
1020/1088
 (92-95)	
Cut-off 200
N
(CI)	40,1%
59/147
 (32-49)	94,7%
1030/1088
 (93-96)	46,3%
68/147
 (38-55)	92,6%
1008/1088
 (91-94)	34,7%
51/147
 (27-43)	97,4%
1060/1088
 (96-98)	38,8%
57/147
 (31-47)	95,7%
1041/1088
 (94-97)	
	Test characteristics for different cut-off values (ng/ml) in three strategies of double FIT sampling, in comparison to single FIT sampling for detection of colorectal cancer in 1235* individuals referred for colonoscopy (screen relevant neoplasia in 147, no AA nor CRC in 1088). *16 cases of late stage CRC were excluded since they were not considered screen relevant. 	
	FIT = faecal immunochemical test, “one of two FITs+” = at least one of both FITs above the cut-off value, “two of two FITs+” = both FITs above the cut-off value, “mean of two FITs+” = geometric mean of both FITs above the cut-off value, CI = confidence interval, sens = sensitivity, spec = specificity

Figure III. ROC curves of single and double FIT sampling strategies for the detection of screen relevant neoplasia, including 251 cases with unsure date of FIT sampling.

		FIT = faecal immunochemical test, “one of two FITs+” = at least one of both FITs above the cut-off value, “two of two FITs+” = both FITs above the cut-off value, “mean of two FITs+” = geometric mean of both FITs above the cut-off value, AUC = area under the curve, CI = confidence interval 

Table VI. Comparison of sensitivity of single and double FIT sampling for screen relevant neoplasia, at fixed specificities, including 251 cases with unsure date of FIT sampling.
	Single FIT		“one of two FITs+”			“two of two FITs+”			“mean of two FITs+”			
Spec	Sens	Cut-off	Sens	Cut-off	p-value	Sens	Cut-off	p-value	Sens	Cut-off	p-value	
85%	55,1%	35	57,1%	66	0,791	n.a.*	n.a.*	n.a.*	57,6%	41	0,791	
90%	49,0%	74	55,1%	112	0,039	47,6%	29	0,727	52,4%	66	0,289	
95%	38,1%	240	40,1%	403	0,774	42,2%	93	0,180	40,1%	170	0,549	

		Corresponding cut-off values (ng/ml) and sensitivities for screen relevant neoplasia of single FIT sampling and three different strategies of double FIT sampling at fixed specificities.	
		FIT = faecal immunochemical test, “one of two FITs+” = at least one of both FITs above the cut-off value, “two of two FITs+” = both FITs above the cut-off value, “mean of two FITs+” = geometric mean of both FITs above the cut-off value, spec = specificity, sens = sensitivity, cut-off = cut-off value,  n.a.* = no corresponding sensitivity and cut-off value found in the range 50-200ng/ml
	 
